# Supplementary material for: Identification of pathogenic missense mutations using protein stability predictors
Source: Sci Rep. 2020 Sep 21;10:15387. doi: 10.1038/s41598-020-72404-w (PMC7506547; doi:10.1038/s41598-020-72404-w)
Supplement: Supplementary file 1 — Supplementary Information 1. [file 41598_2020_72404_MOESM1_ESM.pdf]

Supplementary Material for 'Identification of pathogenic missense mutations using protein stability predictors' by Lukas Gerasimavicius, Xin Liu and Joseph A Marsh, *MRC Human Genetics Unit, Institute of Genetics and Molecular Medicine, University of Edinburgh, Edinburgh EH4 2XU, UK*

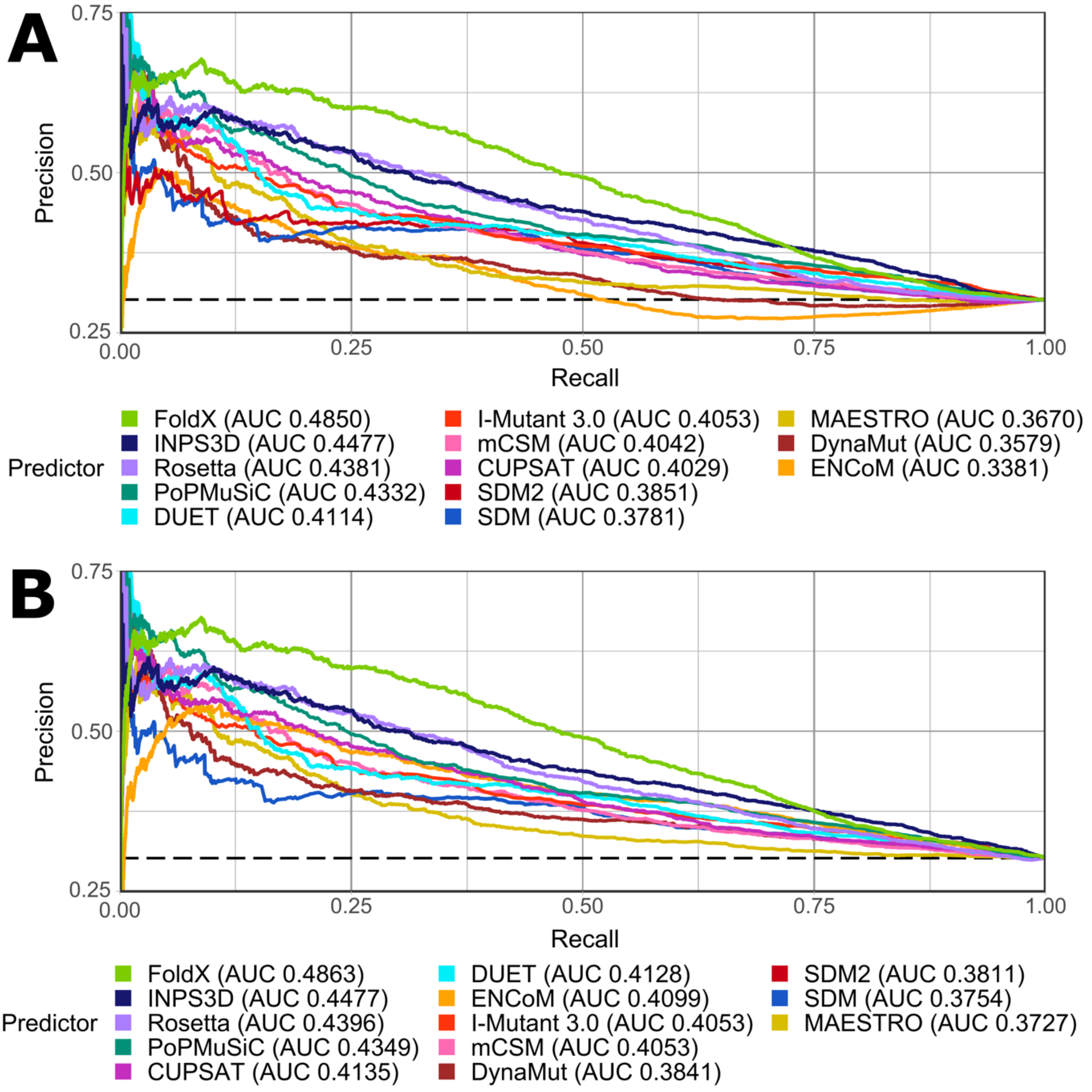

Figure S1. Precision-recall analysis of predicted ΔΔG values. Precision-recall (PR) curves are plotted for each predictor, with the classification performance being presented next to its name in the form of area under the curve (AUC)**.** The horizontal dashed line represents the baseline lowest performance of a predictor, derived from the two-class balance of the dataset, and here corresponds to ~0.3018. Due to the nature of PR analysis a downsized dataset was employed which contained only variants with no missing values for any predictor. **A)** ROC curves for classification performance using raw ΔΔG value scale for each predictor**. B)** ROC curves for predictor classification performance when using absolute ΔΔG values**.** The figure was generated in R v3.6.3 (https://www.r-project.org) using ggplot2 v3.3.0 (https://ggplot2.tidyverse.org/), both freely available.

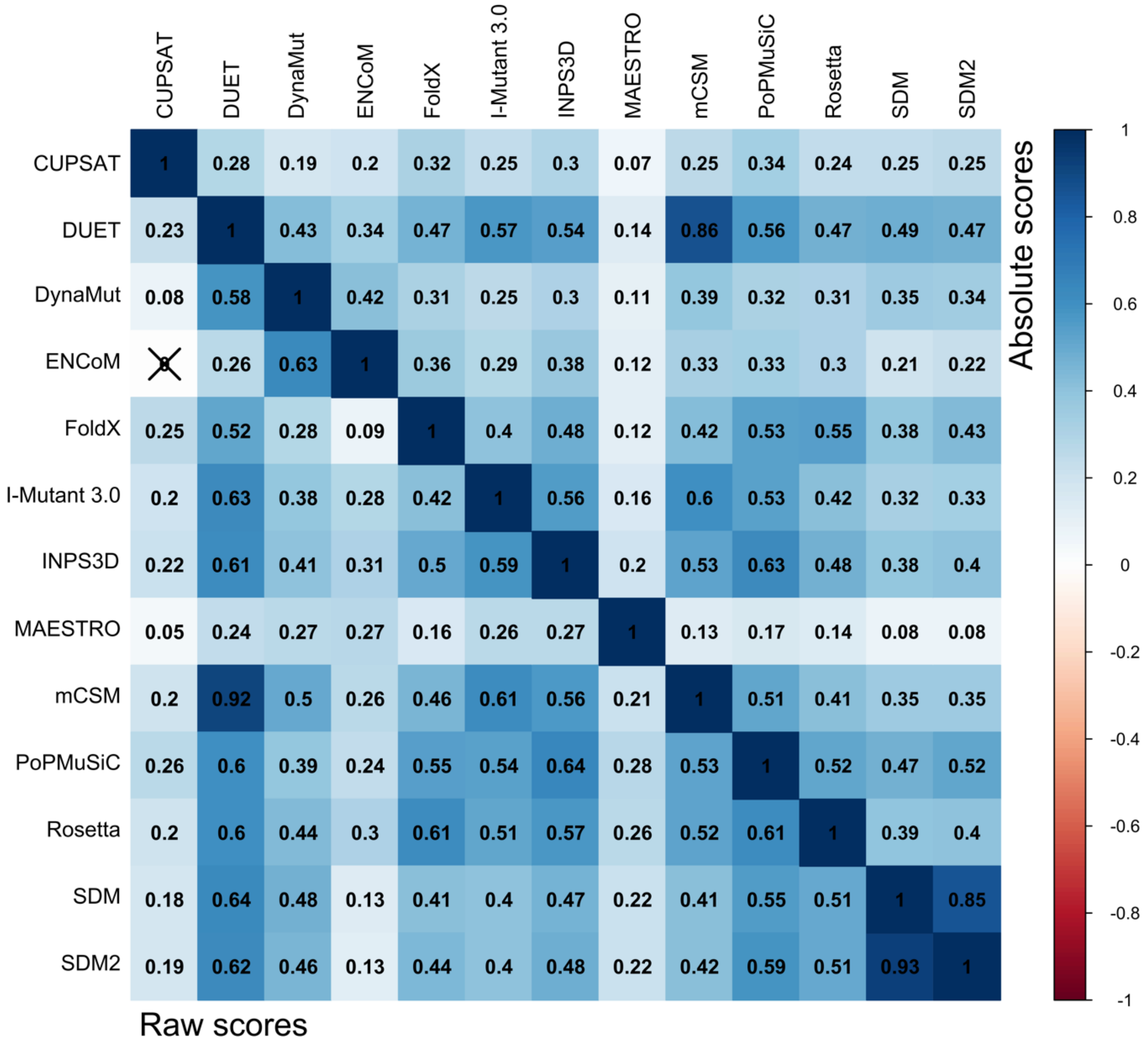

Figure S2. Spearman rank correlation of predicted raw and absolute ΔΔG values between different stability prediction methods. The lower and upper triangles of the matrix represent raw and absolute ΔΔG value rank correlation. Crossed-out values indicate insignificant correlation. The figure was generated in R v3.6.3 (https://www.r-project.org) using corrplot v0.84 (https://github.com/taiyun/corrplot), both freely available.

Table S1. Evaluation of predictor performance using raw and absolute ΔΔG values on the downsized missense variant dataset. DeLong approximation was used to derive the 95% confidence intervals for all the predictors. The dataset used was the same as for the precision-recall analysis, and contained no missing values for any predictors.

Predictor	AUC from raw ΔΔG (95% confidence interval)	AUC from abs. ΔΔG (95% confidence interval)
FoldX	0.658–0.683	0.664–0.688
INPS3D	0.644–0.668	0.647–0.671
Rosetta	0.611–0.636	0.621–0.646
PoPMuSiC	0.617–0.641	0.621–0.646
CUPSAT	0.574–0.600	0.596–0.622
MAESTRO	0.533–0.559	0.544–0.569
SDM	0.574–0.600	0.575–0.600
SDM2	0.583–0.609	0.581–0.606
mCSM	0.579–0.605	0.582–0.608
DUET	0.595–0.620	0.599–0.623
I-Mutant 3.0	0.602–0.626	0.602–0.627
ENCoM	0.482–0.510	0.616–0.640
DynaMut	0.515–542	0.573–0.598

Table S2. Pathogenic and putatively benign missense variants used in this study, along with structures used and outputs from all predictors.

Provided as a separate file 'TableS2.xlsx'
